# Proceedings Medical Association of Nebraska

**Published:** 1874-01

**Authors:** 


We have received the proceedings of the Nebraska State Medical Society, at the fifth annual session, held in Nebraska City, June 3d and 4th, 1873.

The committee on correspondence acknowledge the reception of the proceedings of the Georgia State Medical Association and return thanks. Dr. Geo. Tilden, of Omaha, reported a case of *idiopathic cerebro-spinal meningitis*, successfully treated with repeated doses of *calomel.* Dr. V. H. Coffman reported a case of ovariotomy with fatal termination on the fifth day.

Dr. L. H. Robbins reports a case of *lasceration and loss of one-fourth of the scalp*, in a man of fifty years, with good recovery.

Dr. Coffman reported a case of *cyst in posterior cul-de-sac of*

*vagina.* Cyst opened and found not to communicate with the peritoneal cavity. Good recovery.

Dr. Robbins relates a very remarkable case of *extra uterine pregnancy.* Patient conceived in 1863, in Kentucky. When, on the 10th of February, 1864, a partial operation for ovarian pregnancy was performed. "An incision cutting across the oblique muscle, was made over the middle of the tumor, and the *head* of the fœtus removed, but from some reason unknown to me, the operation was not completed, although the patient said the Doctors were alarmed at the hemorrhage, and abandoned the removal of the entire fœtus." She afterwards suffered with "labor pains," great numbers of small spicula of bone were removed from the vagina, and, "strange and incredible as it must appear, the small spicula of fœtal bones were ejected in vomiting, as well as per vaginam." At this time the patient was attacked with pneumonia, expectorating freely. "An examination of the sputa revealed several fine, sharp spicula of bone, about as fine and sharp as the smallest cambric needle." The patient recovered.

Dr. James A. Peabody, of Omaha, reports a case of *double placenta, death from heart clot;* nine cases of *metro-peritonitis,* and case of *fibroid tumor of uterus.*

Dr. A. Bowen, the President, addressed the Society upon *vinous, spirituous and malt liquors as therapeutical agents.*

The next annual meeting takes place in Omaha, on the first Monday in June. The pamphlet is neatly gotten up, but, we regret to say, the proof-reading is *very* defective.

